# Flat-Lattice-CNN: A model for Chinese medical-named-entity recognition

**DOI:** 10.1371/journal.pone.0331464

**Published:** 2025-09-18

**Authors:** Shanshan Wang, Kunyuan Zhang, Ao Liu

**Affiliations:** 1 School of Economics and Management, Yan’an University, Yan’an, China; 2 College of Computer Science, Inner Mongolia University, Hohhot, China; 3 School of Mathematics and Computer Science, Yan’an University, Yan’an, China; University 20 Aout 1955 skikda, Algeria, ALGERIA

## Abstract

**Background:**

In the ﬁeld of internet-based healthcare, the complexity of pathology features across various disciplines, coupled with the lack of medical training among most patients, results in medical named entities in doctor patient dialogue texts exhibiting long and multiword syntactic patterns, posing new challenges to named-entity recognition algorithms.

**Methods:**

To address the issue mentioned above, in this study we integrate Convolutional Neural Networks (CNNs) of diﬀerent dilation rates on top of Flat-Lattice architecture to construct the Flat-Lattice-CNN model. This model not only considers the semantic information of characters and words, as well as their absolute and relative positional information, but also extracts multiple-token co-occurrence relationship features among characters/words spanning diﬀerent distances to improve the recognition accuracy of long medical-named entities.

**Results:**

Experimental results show an improved performance in the task of recognizing medical-named entities on all evaluation datasets, especially on CTDD with a 2.3% increase in F1 score. The proposed Flat-Lattice-CNN model eﬀectively addresses the challenges posed by long and multiword syntactic patterns in medical-named entities, oﬀering improved recognition accuracy and demonstrating the potential for enhancing medical-named-entity recognition in internet-based healthcare dialogues.

## 1 Introduction

Named Entity Recognition (NER) is one of the fundamental tasks in Natural Language Processing (NLP), with its primary function being to identify speciﬁc concept entities in a text. NER is crucial for areas such as machine translation, information extraction, and question-answering systems. Medical-named-entity recognition aims to extract key information from massive amounts of unstructured medical data, such as drugs, surgeries, symptoms, body parts, and diseases, providing foundational support for the development of medical research and the widespread adoption of smart healthcare systems [1]. In recent years, with continuous advancements in information technology and an increased awareness of service quality, online healthcare has gradually become an important supplement to traditional face-to-face healthcare [2]. On internet-based healthcare platforms, doctor patient communication during consultations is often recorded and saved in text form. However, due to the lack of medical training among most patients and the complexity of pathological features across different medical disciplines, patients often use lengthy expressions to describe their conditions and medical histories. Consequently, related entities tend to have long and multiword names. For example, in the ﬁeld of obstetrics and gynecology, the sequence “月经过后老是再沥沥拉拉一周左右褐色的分泌物”(Continuous brown discharge for about a week after menstruation) represents a lengthy and multiword medical symptom entity [3]. Another illustration of this is“发烧” (fever), which is always considered a complete and independent medical-symptom entity, as is “间歇夜间发热” (Intermittent fever during the night). Although the two terms may correspond to diﬀerent pathological conditions, extant research often treats them equivalently. Speciﬁcally, in the phrase “间歇夜间发热”(Intermittent fever during the night) the term “间歇夜间”(intermittent) is frequently overlooked in labeling and recognition [4].

Currently, research on medical text NER mainly focuses on electronic medical records, medical literature, and medical books, with relatively limited attention to online healthcare-dialogue texts [5]. Although existing methods such as BiLSTM-CRF [6], Bert-CRF [7], and LeBert-CRF [8] have achieved eﬀective results in the ﬁeld of medical NER, they face certain limitations when applied to Chinese online healthcare-dialogue texts. The speciﬁc reasons for these limitations follow: First, medical personnel primarily developed these methods for recognizing shorter named entities, whereas medical-named entities in doctor patient dialogues on online healthcare platforms exhibit long and multiword syntactic characteristics; Second, most models focus on English texts, but the syntactic features of English and Chinese diﬀer signiﬁcantly. English is character-based, whereas Chinese relies heavily on word-level features in addition to character features; finally, scholars common formalize NER as a sequence labeling problem, it essentially involves modeling the relationships among words regarding an entity [9]. So far, algorithms (such as attention mechanisms) [10] essentially capture pairwise relationship information between tokens (characters or words), and an eﬀective mechanism for capturing multiple-token co-occurrence dependency features of long and multiword-named entities is still lacking.

To address these issues, in this study we propose a new model integrating the Flat-Lattice architecture and multigranularity dilated-convolution network: Flat-Lattice-CNN, for NER in internet-based doctor patient dialogue texts. The Flat-Lattice mechanism can extract long-distance dependency relationships between tokens (characters or words) in an entity, however it is mainly limited to capture pairwise relationship information between tokens. Therefore, to model the co-occurrence-dependency features of long multiple-word entities, we integrate a multigranularity dilated-convolution networks. Experimental results show that this model generally improves performance in the medical NER task in datasets.

The main contributions of this paper follow: First, in this paper we aim to address the relatively underexplored issue of long NER. Second, the proposed Flat-Lattice-CNN model appears to alleviate some of the challenges posed by long and multiword syntactic structures in medical-named entities, contributing to an improvement in recognition accuracy. Third, we emphasizes that, in addition to the pairwise dependencies between characters or words, multitoken co-occurrence dependencies also exist within sentences. Although attention mechanism can effectively capture the former, use of CNNs can improve the ability to model the later. In this study, CNNs serve as a valuable complement to attention mechanisms in learning contextual features.

The remainder of this paper is organized as follows: in Section 1, we provide the introduction; In Section 2, the related work is reviewed. Section 3 presents the proposed Flat-Lattice-CNN model. In Section 4 the experimental results are provided, Section 5 offers a detailed discussion, and Section 6 concludes the paper.

## 2 Related work

### 2.1 Named entity recognition

NER research has evolved over the years from initial ﬂat NER to nested NER and to discontinuous NER [9,11]. Compared to general-domain NER, medical NER tasks require the incorporation of the unique linguistic characteristics and semantic knowledge of the medical discipline. A review of the literature reveals that in most existing work in medical NER, researchers focused mostly on input-representation learning. For instance, Li et al. used Lattice LSTM to integrate lexical information and learned contextual information from electronic medical records based on ELMo [12]. Luo et al. [6] used string-matching methods to pair disease dictionaries with characters and proposed a BiLSTM-CRF model combined with a dictionary- attention layer. Another study [2] proposed a BERT-BiLSTM-CRF model that integrates lexical and root features, using the BERT model to enhance the contextual semantic information of Chinese clinical records. Additionally, researchers focused on nested entity recognition in medical texts [13]. Recently, scholars noted the phenomenon of discontinuous named entities in medical texts, such as “腿和胳膊疼”(pain in legs and arms). To address this issue, in related research, scholars introduced new label tags, such as “感觉(疼)”(feeling (pain)) to handle such discontinuous entities. The number of these tags needs to be updated and enriched according to the corpus [14]. We used the models described in the aforementioned literature as baselines for experimental comparison with the approach proposed in this study.

In addition to the studies described above, Huang’s work contributes by framing the NER as a language-modeling task, aiming to address the issue of label mismatch between the source and target domains in transfer learning [15]. Cui’s work shares similarities with Huang’s approach [16]. Yu’s study focuses on leveraging image information to assist in NER [17]. To the best of our knowledge, researchers have rarely discussed long-named entities in the literature to date. Newmann-Griffi [18] previously defined entities consisting of more than 10 tokens as “very long entities”.

### 2.2 Lattice structures in NER tasks

Compared to English, NER in Chinese is more challenging due to word segmentation. Recent studies demonstrated that lattice structures have advantages in using word information and avoiding the propagation of segmentation errors. In a lattice structure, users can match a sentence with a dictionary to obtain potential words that may be crucial for NER. Speciﬁcally, in the lattice, each node represents a token (either a character or a potential word), with the position of a word determined by its ﬁrst and last character rather than being sequentially arranged. Lattice-based models use attention mechanisms to integrate a variable number of word nodes at each position. However, due to the dynamic nature of this lattice structure, related methods cannot fully leverage graphics-processing-unit parallel computing. To address this, scholars proposed the Flat-Lattice model. This model converts the lattice structure into a ﬂat-lattice, based on transformer architecture, using designed head and tail positional encodings to losslessly reconstruct the lattice structure [5].

### 2.3 CNN

CNN [19] is a specialized type of artiﬁcial neural network focused in areas such as video classiﬁcation, image recognition, image captioning, facial detection, information extraction, text processing, and speech recognition. Typically, a CNN model is composed of an input layer, convolutional layers, pooling layers, activation layers, and, at the top, fully connected layers and a loss-function layer. The purpose of the convolutional layers is to extract various features: lower convolutional layers capture low-level features whereas higher convolutional layers extract high-level features. The dilation rate controls the receptive ﬁeld of the convolutional kernels; a higher dilation rate increases the receptive ﬁeld. In text processing, CNNs allow the model to capture long-range dependencies and multiword co-occurrence features.

## 3 The proposed model

**Fig 1:**
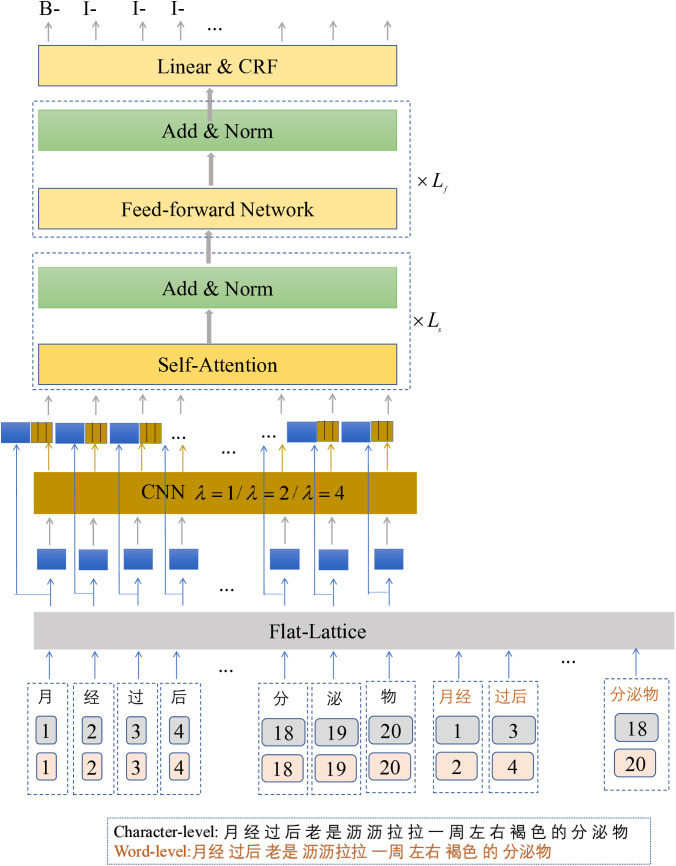
The architecture diagram of the FLAT-Lattice-CNN model.

The proposed model Flat-Lattice-CNN comprises three primary components: the input layer (a combination of Flat-Lattice and CNN), the Self-Attention and Feed-Forward Network (FFN) layer, and the label prediction layer (CRF), illustrated in Fig 1.

First, the Flat-Lattice is used to obtain the embeddings of the tokens (characters or words) in the input sentence. Building upon this, the model employs convolutional kernels with varying dilation rates to capture the features of multiword co-occurrence dependencies at different distances, transforming them into vector representations. These vectors are then concatenated to generate the final embedding representation for each character. Each character in the sequence encodes the semantic information of the word, whereas the word itself does not provide input to the downstream model.

Second, the embedding vectors of the characters (the word itself does not provide input to the downstream model) from the input layer pass into the Self-Attention and FFN layer, where they employ Self-Attention and FFN mechanisms to learn the contextual dependencies of the characters. The protocol subsequently applies a residual structure to extract the final semantic features. Finally, the model further inputs these semantic features into the label-prediction layer, using CRF to predict the labels. The following sections will provide a detailed explanation of each component.

### 3.1 The Flat-Lattice model

The Flat-Lattice model initially flattens the lattice structure, composed of characters and their corresponding words, into a sequence of spans. Subsequently, the model integrates positional information, enabling the derivation of embedding vectors for each token. Each span corresponds to a token (either a character or a word) and encapsulates positional information for the head and the tail, as shown in the dashed box in Fig 1. The head and tail denote the positions of the first and last characters of the token in the original sequence, reflecting its spatial position in the lattice structure.

The model assigns each token (character or word) two positional indices: head position and tail position. These positional indices allow reconstruction of the lattice from a sequence of tokens. Three possible relationships emerged between any two spans $x_{i}$ and $x_{j}$ in the input sequence: intersecting, containing, and separating, determined by the head and tail of the spans. This positional mechanism can simultaneously represent both absolute and relative positional information of two tokens. Equation 1 calculated the relative position of any two spans $x_{i}$ and $x_{j}$.


$$\begin{array}{c} d_{ij}^{\left(hh\right)}=head[i]-head[jnonumber\\ d_{ij}^{\left(ht\right)}=head[i]-tail[j]\\ d_{ij}^{\left(th\right)}=tail[i]-head[j]\\ d_{ij}^{\left(tt\right)}=tail[i]-tail[j] \end{array}$$ (1)

where *d* represents the distance between heads, and *i* and *j* have similar meanings.

The relative positional encoding of a SPAN is a simple nonlinear transformation of four distances, calculated as in Equation 2:


$$R_{ij}=ReLU\mathrm{\backslash nolimits}(W_{r}(P_{d_{ij}^{\left(hh\right)}}⊕P_{d_{ij}^{\left(th\right)}}⊕P_{d_{ij}^{\left(ht\right)}}⊕P_{d_{ij}^{\left(tt\right)}}))$$ (2)

where $W_{r}$ are learnable parameters, ⨁ denotes the concatenation operator, and Vaswani et al.calculated $P_{d}$ [8], which can be computed using Equation 3:


$$\begin{array}{c} P_{d}^{\left(2k\right)}=\sin(d/10000^{2k/d_{Model}})\\ P_{d}^{\left(2k+1\right)}=\cos(d/10000^{2k/d_{Model}}) \end{array}$$ (3)

Within which, *d* and *k* denote the dimension indices of the positional encoding.

Finally, the protocol employs a variant of Self-attention to incorporate the relative positional encoding of SPANs, thereby enhancing the model’s capacity to capture sequence-level information. The Flat-Lattice derives the embedding vector $W_{F}$ for each span in the input sequence.

### 3.2 Dilated convolution CNN

Upon the aforementioned embedding vectors $W_{F}$, in this study we introduce convolutional mechanisms with varying dilation rates to capture a multiword co-occurrence dependency feature vectors $W_{c}$ across different distances. It then concatenates $W_{F}$ and $W_{C}$ to form the ﬁnal input embedding vector $\left[W_{F};W_{C}\right]$. As shown in Equation (4), the convolution mechanism features a local receptive ﬁeld and translation invariance. The local receptive ﬁeld allows the mechanism to focus on local feature extraction without considering the complexity of the entire input, whereas translation invariance means that the recognition of features is unaﬀected by their absolute position. The Dilated-Convolution CNN structure includes the following key components:

1. Basic Convolution Layer: Initially, a one-dimensional convolution layer with an optional dropout rate extracts features from the input. This layer helps map the input data into a higher dimensional representation space and performs nonlinear transformations through the Rectified Linear Unit activation function. In this step, we aim to provide a rich input representation for the subsequent multigranularity-dilated-convolution layers.
2. Multigranularity-Dilated Convolution: To capture the co-occurrence dependency information of multiple words, we introduce multiple convolution kernels with diﬀerent dilation rates. Each convolution kernel has its own receptive ﬁeld size, enabling it to eﬀectively capture semantic information within diﬀerent ranges. Speciﬁcally, assuming we use a variable λ to measure the dilation rate of the dilated convolution, the relationship between the actual convolution kernel size after dilation and the original convolution kernel size appears in Equation 4:

K=k+(k−1)(λ−1) (4)

Here, *k* represents the original convolution kernel size, λ denotes the convolution dilation rate; *K* stands for the actual convolution kernel size after expansion. Equation 5 shows the calculation for the size after convolution:


$$W=\frac{w-k+2p}{s}+1=samll\;\frac{w\;-k\;-(k\;-1)(\lambda -1)+2p}{s}+1$$ (5)

where *w* is the input size, *k* is the original convolution kernel size, λ is the dilation rate, *s* is the stride, and *p* is the padding.

3. Residual Connections [20]: To ensure the ﬂow of information and preserve important information from the original input, a strategy of residual connections is employed after the multigranularity-dilated convolutions.

### 3.3 Self-attention and FFN

In this study, integrating Flat-Lattice and multi-dilation-rate convolution mechanisms, we obtain the embedded representations of the input sequence. These embedded sequences are then fed into the Self-Attention and FFN layers, enhancing the model’s ability to consider the contextual information of the entire sequence for each element, thereby better understanding the relationships between elements and other parts of the sequence.

The Attention mechanism [10], often referenced as scaled dot-product attention, is a core methodology in NLP and deep learning, playing a crucial role in a wide range of tasks, including machine translation, text summarization, and sentiment analysis. The Attention mechanism allows models to dynamically assign different levels of importance to various segments of an input sequence, thereby facilitating the modeling of dependencies between tokens. The attention function is formulated as follow.


$$Attention(Q,K,V)=softmax(\frac{QK^{T}}{\sqrt{d_{k}}})V$$ (6)

At the core of the attention function are three key components: the Query (Q), Key (K), and Value (*V*) vectors. Dimensions of *Q* and *K* are $d_{k}$. The Query *Q* represents the aspect of the sequence that is the focus of the model. The Key *K* assists in identifying and locating important elements within the sequence, whereas the Value *V* contains the information to be considered. The attention methodology illustrated in Equation (1) uses *QK*^*T*^ as weights to perform a weighed aggregation of value vectors.

An FFN is one of the simplest and most commonly employed types of artificial neural networks. It comprises of layers of neurons where information flows unidirectionally—from input to output—without the presence of cycles or loops. Characterized by its hierarchical architecture, an FFN typically includes an input layer, one or more hidden layers, and an output layer. Each neuron in a given layer fully connects to every neuron in the subsequent layer, with no feedback connections. This structure makes FFNs particularly well-suited for supervised learning tasks.

### 3.4 Prediction layer

The label-prediction layer employs a CRF model [21], a discriminative undirected graphical model that achieves globally optimal label sequences by studying the relationships between labels. The probability of a particular label sequence y is a normalized product of potential functions, given observation sequence x.


$$\exp(\sum_{j}\lambda_{j}t_{j}(y_{i-1},y_{i},x,i)+\sum_{k}\mu_{k}s_{k}(y_{i},x,i))$$ (7)

Where $t_{j}(y_{i-1},y_{i},x,i)$ is a transition feature function of the entire observation sequence and the labels at positions *i* and *i – 1* in the label sequence; $s_{k}(y_{i},x,i)$ is a state feature function of the label at position *i* and the observation sequence; and $\lambda_{i}$ and $\mu_{k}$ are parameters to be estimated from training data.

## 4 Experimental results and analysis

### 4.1 Experimental data

To validate the eﬀectiveness of the proposed model in Chinese NER tasks, we conducted experiments on two public Chinese medical NER datasets, IMCS-2021 [22] and CTDD [3]. These datasets are described in Table 1. CTDD is a Chinese dataset of internet-based doctor patient dialogues in the discipline of gynecology and obstetrics, whereas IMCS-NER is a Chinese dataset of online doctor-patient dialogues in pediatrics. As shown in Table 1, the average length of entities in CTDD is 4.33, whereas it is 2.62 in IMCS-2021. Thus, we can conclude that the proportion of long entities in CTDD is higher than in IMCS-2021. In the CTDD dataset, the proportion of very long entities is 3.9%, whereas in the IMCS-2021 dataset, this proportion is 0.02%. This finding indicates that, compared to the IMCS-2021 dataset, the CTDD dataset can be considered, to some extent, a dataset containing long-named entities.

**Table 1:** Descriptive information for the CTDD dataset and IMCS-NER dataset.

	CTDD	IMCS-NER
Number of all named entities	63560	74698
Average length of entities	4.33	2.62
Total characters	1,700,392	1,621,161
Ratio of tagged characters to total characters	16.2%	12.1%
Average word count of dialogues	713.55	589.04
Ratio of very long entities to total entities	3.9%	0.02%

**Fig 2:**
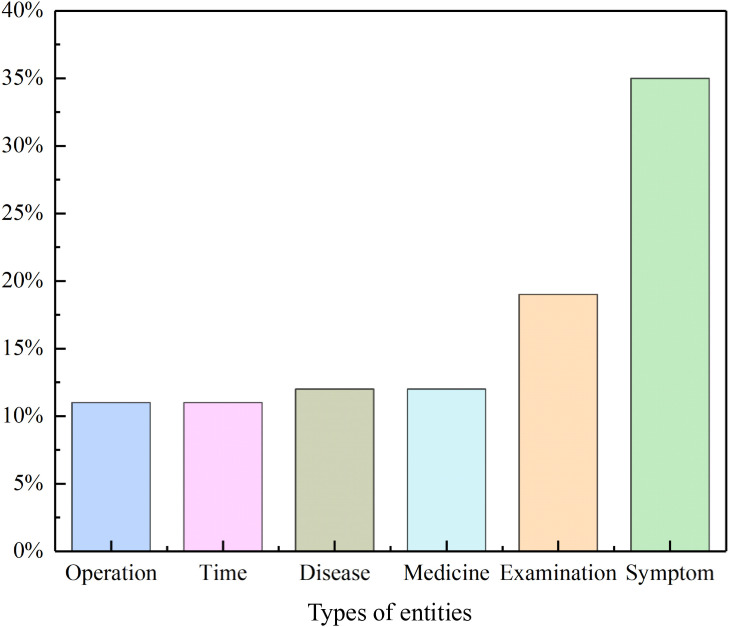
Distribution of entity types in the CTDD dataset.

The IMCS-2021 dataset covers ﬁve types of entities, including symptoms, examinations, and medication names. In this study, we randomly selected 73,603 samples as the training set, 12,517 samples as the validation set, and 12,332 samples as the test set. The CTDD dataset contains six types of entities, such as time, diseases, and symptoms. From this dataset, 21,633 samples were randomly selected as the training set, 3,757 samples as the validation set, and 4,847 samples as the test set. The distribution of entity types in the CTDD dataset appear in Fig 2.

### Entity types

The parameter settings for the NER experimental evaluation in this study appear in Table 2.

**Table 2:** Parameterization.

Parameters	Quantities
Learning rate	6e-4
Batch	10
Epoch	50
Number of attention heads	8
Dimensions of the Attention Head	20
Input dimension	160
Channel number	160
Dropout	0.5

### 4.2 Evaluation index

The experimental results in this study were evaluated using the F1-score, calculated as shown in Equation 7:


$$\begin{array}{c} P=\frac{Predicting\;the\;correct\;set\;of\;entities}{Total\;number\;of\;entities\;obtained}\times 100\%\mathrm{\backslash vspace}10pt\\ R=\frac{Predicting\;the\;correct\;set\;of\;entities}{Number\;of\;data\;set\;entities}\times 100\%\mathrm{\backslash vspace}10pt\\ F_{1}=\frac{2\;\times P\;\times R}{P+R}\times 100\% \end{array}$$ (8)

### 4.3 Baseline model

(1) The BiLSTM-CRF model [6] employs a BiLSTM network to capture the contextual information in input sequences, and combines this with a CRF to model the sequence-labeling problem.
(2) The Bert-CRF model [7] integrates the pretrained BERT model with a CRF. This model leverages the powerful contextual understanding capabilities of BERT to perform representation learning on input sequences, and uses the CRF layer for global optimization of the label sequences.
(3) The LeBert-CRF model [8] builds on the Bert-CRF model by incorporating domain-speciﬁc pre-training methods to enhance its performance.
(4) The Lattice LSTM model [12] is a neural network model based on a lattice structure. It introduces character-level information at the model’s input and, through the design of the lattice structure, enables the model to better capture the dependencies among characters within words.

### 4.4 Analysis of experimental results

To better observe the eﬀectiveness of the proposed model for Chinese medical NER, experiments have been conducted using BiLSTM-CRF, Lattice-LSTM, Bert-CRF, LeBert-CRF, and FLAT-Transformer models as control groups on two public Chinese medical NER datasets, IMCS-2021 and CTDD. The parameters for the control-group models were set to their default values. The performance of diﬀerent models on Chinese NER tasks appears in Table 3. Although long and multiword entities are characteristic of medical-domain texts, other domains may also have long entities. Therefore, we conducted comparative experimental evaluations on the Weibo Chinese dataset [23] using the FLAT-Transformer and Flat-Lattice-CNN algorithms, as shown in Table 3. From Table 3, we observe that the proposed model shows a general performance improvement. Specifically, on datasets with long entities, the F1-score increased by 2%, which, to some extent, demonstrates the eﬀectiveness of the proposed model.

**Table 3:** The experimental results on the CTDD, IMCS, and Weibo datasets.

	CTDD			IMCS-2021			weibo		
Model	P(%)	R(%)	F1(%)	P(%)	R(%)	F1(%)	P(%)	R(%)	F1(%)
Bert-CRF	53.09	52.75	52.92	88.46	92.35	90.37	–	–	–
LeBert-CRF	52.63	54.25	53.42	86.53	92.91	89.60	–	–	–
BiLSTM-CRF	46.26	50.17	48.21	85.67	88.72	87.61	–	–	–
Lattice LSTM	**57.14**	55.14	56.14	89.37	90.43	90.10	53.04	62.25	58.79
FLAT-Lattice Transformer	53.62	56.47	55.14	88.83	93.43	91.07	69.84	77.37	73.41
LLama	36.23	41.67	38.76	42.62	44.71	39.81	42.30	39.71	36.20
Flat-Lattice-CNN (ours)	55.63	**59.10**	**57.31**	**89.44**	**93.51**	**91.43**	**70.29**	**79.69**	**74.69**

**Fig 3:**
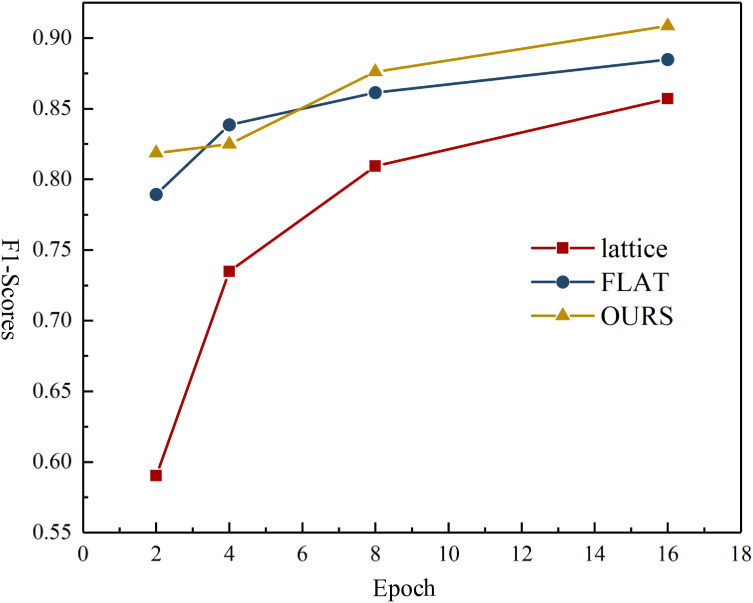
The inference efficiency across models.

Given that the proposed model uses convolutional layers to capture multitoken co-occurrence-dependency features within entities, our experiments show that the model captures information more quickly. At epoch = 15, the F1 score on the CTDD dataset reached 0.52 (approximately 91.2% of the ﬁnal result), whereas the F1-score for the FLAT-Lattice-Transformer reached 0.48 (approximately 87.2% of the ﬁnal result). The inference-speed comparison results appear in Fig 3.

### 4.5 The effect of multigranularity-dilated convolution

To better observe the impact of multigranularity-dilated convolution on Chinese medical NER, λ were set as the dilation rate, that is, the dilation factor. We established λ = [1], λ = [1, 2], and λ = [1, 2, 4] as control-group models, with other parameters set to default values. The NER performance on the CTDD dataset with diﬀerent dilation rates at epoch = 15 appears in Table 4. As observed Table 4, when the dilation rate λ = [1, 2, 4], both the F1-score and accuracy values mostly improved.

**Table 4:** Comparison of dilation rates.

Dilation(λ)	F1(%)	ACC(%)
[1]	53.40	86.53
[1,2]	54.50	87.61
[1,2,4]	55.35	87.75
[1,2,4,8]	52.77	87.70

Moreover, an example from the IMCS-2021 dataset is provided to illustrate the effectiveness of the proposed model in enhancing the recognition of long entities, as presented in Table 5. The example sentence is “化验 E 台病毒抗体, 看是否是 eb 病毒感染(test for E-type viral antibodies to determine whether there is an EB virus infection)”. In the dataset, “E 台病毒抗体” is labeled as an entity of the “Examination” type. When the proposed Flat-Lattice-CNN model (with CNNs having dilation rates of 1, 2, and 4) was applied to the sentence, “E 台病毒抗体” was accurately and fully identified as an Examination entity. However, when the Flat-Lattice-CNN model with only dilation rate of 1 was used to process the same sentence, “E 台” and “抗体” were separately recognized as two short Examination entities. Lattice LSTM also classified “E 台” and “病毒抗体” as two distinct short Examination entities. The entities identified by each method shown in Table 5 are denoted by underlining. These findings suggest that the Flat-Lattice-CNN, when employing dilation rates of 1, 2, and 4, demonstrates a potentially improved ability to recognize long entities.

**Table 5:** An example from the IMCS-2021 dataset.

Sentence	化验 E台病毒抗体 (Examination entity), 看是否是 eb 病毒感染
Ours (CNN with λ=1,2,4^)^	化验 **E**台病毒抗体 (Examination entity), 看是否是 eb 病毒感染
Ours (CNN with λ=1^)^	化验 **E**台 (Examination entity) 病毒**抗体** (Examination entity), 看是否是 eb 病毒感染
Lattice LSTM	化验**E**台 (Examination entity) **病毒抗体** (Examination entity), 看是否是 病毒感染

**Fig 4:**
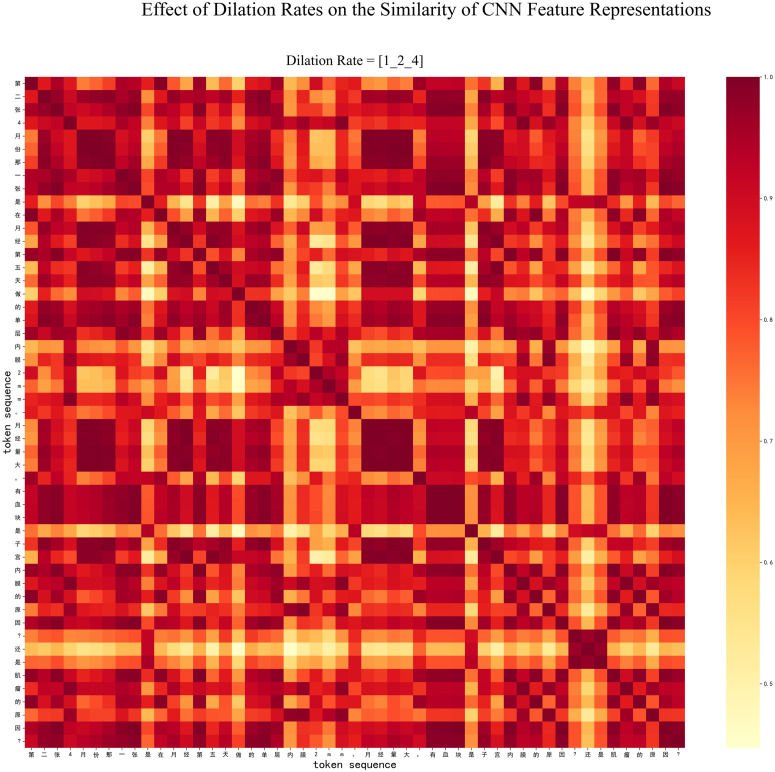
The CNN heatmap for the Flat-Lattice-CNN model with dilation rates of 1, 2, and 4.

**Fig 5:**
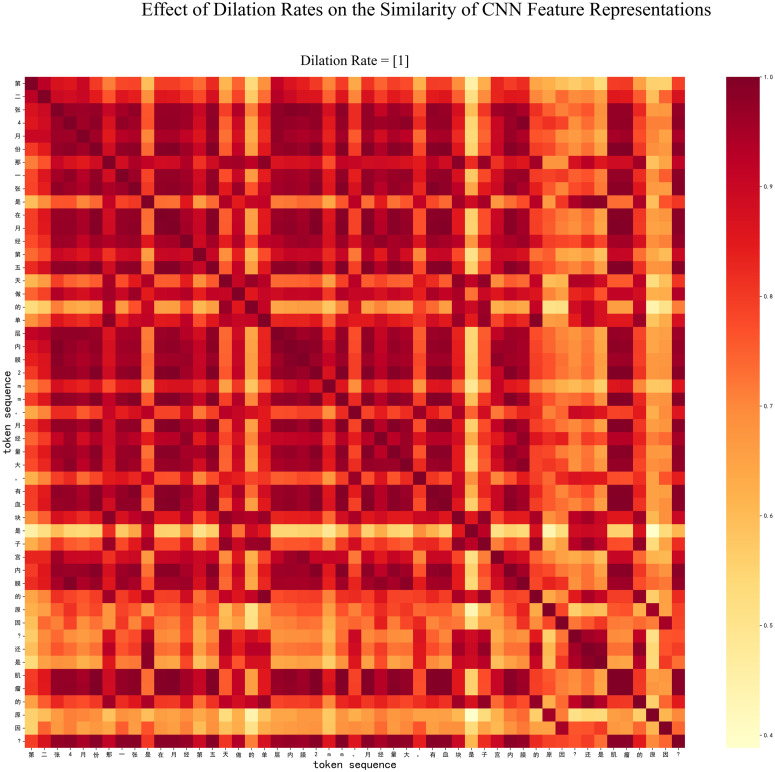
The CNN heatmap for the Flat-Lattice-CNN model with only dilation rate of 1.

Furthermore, take the token sequence “第二张 4 月份那一张是在月经第五天做的单层内膜 2mm。月经量大, 有血块是子宫内膜的原因?还是肌瘤的原因” (The second scan in April was performed on the fifth day of menstruation, revealing a single-layer endometrium with a thickness of 2 mm. The presence of heavy menstrual bleeding and blood clots—do these symptoms originate from the endometrium or uterine fibroids?) as an example for continuing to reveal the effect of multigranularity- dilated convolution. Fig 4 illustrates the impact of convolution strategies with dilation rates of 1, 2, and 4, compared to the model with only a dilation rate of 1 (Fig 5). The heatmap in Fig 4 exhibits a greater distinction between different grid cells, whereas the heatmap in Fig 5 shows a more uniform distribution across cells. The visualization results clearly demonstrate that the model with multi-scale dilation convolutions (1, 2, and 4) outperforms the one with only a dilation rate of 1. This result emerges because this strategy systematically enlarges the receptive field of the convolution layers, enabling the model to capture a broader and more diverse range of local contextual information. As observed from the figures, the model with dilation rates of 1, 2, and 4 not only identifies isolated medical keywords but also establishes stronger semantic connections between clinically relevant concepts that are more spatially distant, such as between “血块(blood clot)” and the more distantly located term “月经 (menstruation)” This result indicates that a more enriched feature representation is beneficial for NER tasks.

### 4.6 The error analysis

According to the definitions from Zhou et al [24], four types of error were analyzed for the Flat-Lattice-CNN model. The proportion of each error type appears in Table 6. From Table 6, we see that the error defined as “Boundary is correct for the entity, but the type is incorrect” accounts for the highest proportion, approximately 91.2%. A possible cause may be label imbalance, where the uneven distribution of labels for different entity types in the training data causes the model to favor more frequently- occurring entity types, leading to mispredictions for rarer entity types. Fig 3 shows that the prevalence of symptom entities in the CTDD dataset is significantly higher than that of other entity types. During error analysis, disease entities were frequently misclassified as symptom entities.

**Table 6:** Error analysis of the Flat-Lattice-CNN model.

Error type	Error definition	Error proportion
Incorrect entity recognition	ldentify non-entities as entities	3%
Entity omissions	Missing the entities in sentence	3.7%
Boundary error	Overlap with the correct entity and entity’s type is correct	2.1%
Entity type error	Boundary is correct for the entity, but the type is incorrect	91.2%

## 5 Discussion

The ambiguous nature of character boundaries in Chinese texts and the syntactic characteristics of named entities in Chinese online medical dialogues drove the design of the Flat-Lattice-CNN model. Unlike English, identifying character boundaries in Chinese texts poses a significant challenge for NLP tasks. Specifically, the semantics of a word formed by the combination of multiple characters can be entirely different from the semantics of the individual characters within it. For instance, “月经” (menstruation) represents a medical entity, whereas the character “月” (month) refers to time, and “经” (pass) refers to something from the past. Therefore, for the task of named-entity recognition in Chinese online-medical-dialogue data, the Flat-Lattice component integrates character-level and word-level semantic information, resulting in more accurate semantic representations.

Moreover, online medical dialogues often involve patients—who lack medical training—describing their conditions using lengthy, multiword expressions, leading to the occurrence of long medical-named entities. Consequently, it is essential to model the dependencies both within these long named entities and between the entities and their broader context. To address this issue, in the present study, we employed dilated CNNs to model these dependencies. Dilated convolutions capture co-occurrence dependencies across multiple tokens, thereby providing a meaningful complement to attention mechanisms, which are mostly designed for pairwise feature learning.

On the whole, the experimental results suggest that the model proposed in this paper is effective. This study makes two primary contributions to the academic field. First, long NER tasks have received limited attention, and this research represents one of the first attempts to address this issue. Second, in contrast to existing NLP studies, this paper proposes that dilated convolutions serve as a valuable complement to attention mechanisms, which primarily capture pairwise dependencies between tokens. This finding is important, as multiword-co-occurrence dependencies also exist among tokens in text.

## 6 Conclusions

This paper provided the Flat-Lattice-CNN model for long NER in Chinese doctor patient dialogue text from internet-based healthcare platforms. Experimental results demonstrate that this model oﬀers improvements in F1-score and convergence speed compared to traditional methods. The main contributions of this paper are summarized as follows: First, we addressed the relatively underresearched issue of long NER. Second, the proposed Flat-Lattice-CNN model alleviates some of the challenges posed by long and multiword syntactic structures in medical-named entities, thereby improving recognition accuracy. Third, this study highlights that, in addition to the pairwise dependencies between characters or words, multicharacter (or multiword) co-occurrence dependencies also exist in sentences. Although the former can be effectively captured by attention mechanisms, the latter is more effectively modeled using CNNs. Study results suggest that CNNs serve as a valuable complement to attention mechanisms in learning contextual features.

In future work, investigation will be conducted on the use of Large Language Models (LLMs) to enhance the efficiency of long NER. Although LLMs have shown promise across various NLP tasks, their performance in domain-specific medical NLP tasks remains suboptimal. Therefore, strategies need to be explored to overcome this limitation.
